# Construction of TUATinsecta database that integrated plant and insect database for screening phytophagous insect metabolic products with medicinal potential

**DOI:** 10.1038/s41598-020-74590-z

**Published:** 2020-10-15

**Authors:** Wakana Nakane, Hisashi Nakamura, Takeru Nakazato, Natsuki Kaminaga, Miho Nakano, Takuma Sakamoto, Maaya Nishiko, Hidemasa Bono, Isao Ogiwara, Yoshikazu Kitano, Kikuo Iwabuchi, Kaoru Kinoshita, Richard J. Simpson, Hiroko Tabunoki

**Affiliations:** 1 Department of Science of Biological Production, Graduate School of Agriculture, Tokyo University of Agriculture and Technology, 3-5-8 Saiwai-cho, Fuchu-shi, Tokyo 183-8509 Japan; 2 Database Center for Life Science (DBCLS), Joint Support-Center for Data Science Research, Research Organization of Information and Systems (ROIS), Mishima, Shizuoka 411-8540 Japan; 3 Department of Pharmacognosy and Phytochemistry, Meiji Pharmaceutical University, 2-522-1 Noshio, Kiyose-shi, Tokyo 204-8588 Japan; 4 Department of United Graduate, School of Agricultural Science, Tokyo University of Agriculture and Technology, 3-5-8 Saiwai-cho, Fuchu-shi, Tokyo 183-8509 Japan; 5 Department of Applied Biological Science, Tokyo University of Agriculture and Technology, 3-5-8 Saiwai-cho, Fuchu-shi, Tokyo 183-8509 Japan; 6 Department of Biochemistry and Genetics, La Trobe Institute for Molecular Science (LIMS), La Trobe University, Melbourne, VIC 3086 Australia; 7 Institute of Global Innovation Research, Tokyo University of Agriculture and Technology, 3-5-8 Saiwai-cho, Fuchu, Tokyo 183-8509 Japan

**Keywords:** Drug discovery, Entomology

## Abstract

Phytophagous insect larvae feed on plants containing secondary metabolic products with biological activity against other predatory organisms. Phytophagous insects can use their specialised metabolic systems to covert these secondary metabolic products into compounds with therapeutic properties useful to mankind. Some Asians drink tea decoctions made from phytophagous insect frass which is believed to be effective against inflammatory diseases. However, insects that can convert plant-derived secondary metabolic products into useful human therapeutic agents remain poorly studied. Here, we constructed the TUATinsecta database by integrating publicly plant/insect datasets for the purpose of selecting insect species. Using TUAT-insecta we selected the Asian swallowtail butterfly, *Papilio xuthus* larvae fed on several species of Rutaceous plants and examined whether the plant-derived secondary metabolites, especially those present in frass, were chemically altered or not. We extracted metabolic products from frass using three organic solvents with different polarities, and evaluated solvent fractions for their cytotoxic effects against several human cell lines. We found that chloroform frass extracts from *P. xuthus* larvae fed on *Poncirus trifoliata* leaves contained significant cytotoxic activity. Our findings demonstrate that screening of insect species using the ‘TUATinsecta’ database provides an important pipeline for discovering novel therapeutic agents that might be useful for mankind.

## Introduction

To date, many medicines have been extracted from natural products. Drugs from natural products comprised 42% of all approved medicines between 1981 and 2014^1^.

Surprisingly, 50% of approved anticancer agents are derived from natural products^1^. For example, paclitaxel, vincristine, and camptothecin are plant-derived anticancer agents obtained from *Taxus brevifolia*^2^, *Catharanthus roseus*^3^, and *Camptotheca acuminate*^4^, respectively. However, resources for plant-derived natural products are being depleted, and in pursuing sufficient resources for natural products, researchers have recognised the need to identify other resources which include secondary metabolites (also referred to as metabolic products) for utilisation as pharmaceutical agents. The majority of resources for natural products are natural plants, many of which are difficult to breed as crops. The need to find new pharmaceutical resources is therefore pressing.

Over one million species of insects are estimated to exist worldwide^5^. Insects have a wide variety of feeding habitats—for example, plant leaves, plant stems, flower nectar, the bodies of trees, tree sap, timber, fruits and the blood, carcasses or excrement of other animals. Each insect species feeds on species-specific food. Insect species which eat only plants are classified phytophagous insects. Secondary metabolites, including those of plants, are known to act as factors that stimulate or inhibit feeding behaviour in insects^6–8^. Some plant secondary metabolites—such as alkaloids—are widely known to have toxic effects on other organisms and cannot be consumed unless they are detoxified. Therefore, the types of plants that insects are able to eat are limited, and most insects have a specific host plant.

Phytophagous insects have evolved a unique system that metabolises secondary metabolites produced by the host plant for escaping food competition^9–11^. Furthermore, they keep the secondary metabolites taken up from the plant in their bodies, and utilise them for escaping from predators^12,13^. In this way, phytophagous insects and plants have a strong coevolutionary relationship. Lepidopteran insects, in particular, may evolve a diverse metabolic process to utilise specific plants. Plant secondary metabolites absorbed into Lepidopteran insect bodies can be converted into other metabolic products with unique structures. For example, swallowtail butterflies use the variety of plants containing herbal substances such as Rutaceae, Apiaceae, Aristolochiaceae, Lauraceae, Magnoliaceae, Annonaceae, or Papaveraceae as their major host plants, converting plant-derived second metabolites into other substances in their bodies^14^. The tobacco hornworm (*Manduca sexta*) and tiger moth (*Arctia caja*) tolerate or even utilise plant secondary metabolites that are toxic to other organisms, to protect against predation or for synthesis of pheromones^15–17^. It is therefore expected that these Lepidopteran insect bodies—and the final metabolites of their frass (also called droppings)—contain plant-derived substances that have changed the chemical structure and biological activity through Lepidopteran insect-specific metabolism.

Two examples of compounds derived from Lepidopteran insects that are useful for humans are papilistatin which has anticancer activity (isolated from *Byasa polyeuctes termessus*^18^) and an analgesic agent, 20-hydroxyecdysone, isolated from the silkworm *Bombyx mori*^18^.

However, compared to the number of studies examining pharmacologically active substances in the insect body, very few studies have examined pharmacologically active substances in insect frass. The insect frass is therefore likely to contain candidates for pharmaceutical agents. It is, however, difficult to find an insect species that excretes frass containing pharmaceutical agents useful for humans and research on using insect frass as pharmaceutical resources is lacking worldwide.

For these reasons, in this study, we constructed a database for choosing insect species that eat plants containing herbal substances or specific secondary metabolites of plants. We then focused on the butterfly *Papilio xuthus*, larvae fed on several species of Rutaceous plants. And we compared the biological activities among each substance which extracted from larval frass fed on *Poncirus trifoliata*, *Zanthoxylum piperitum*, *Citrus natsudaidai*, *Citrus junos* or *Citrus sudachi*.

## Results

### Development of the database “TUATinsecta” for choosing an insect

**Figure 1:**
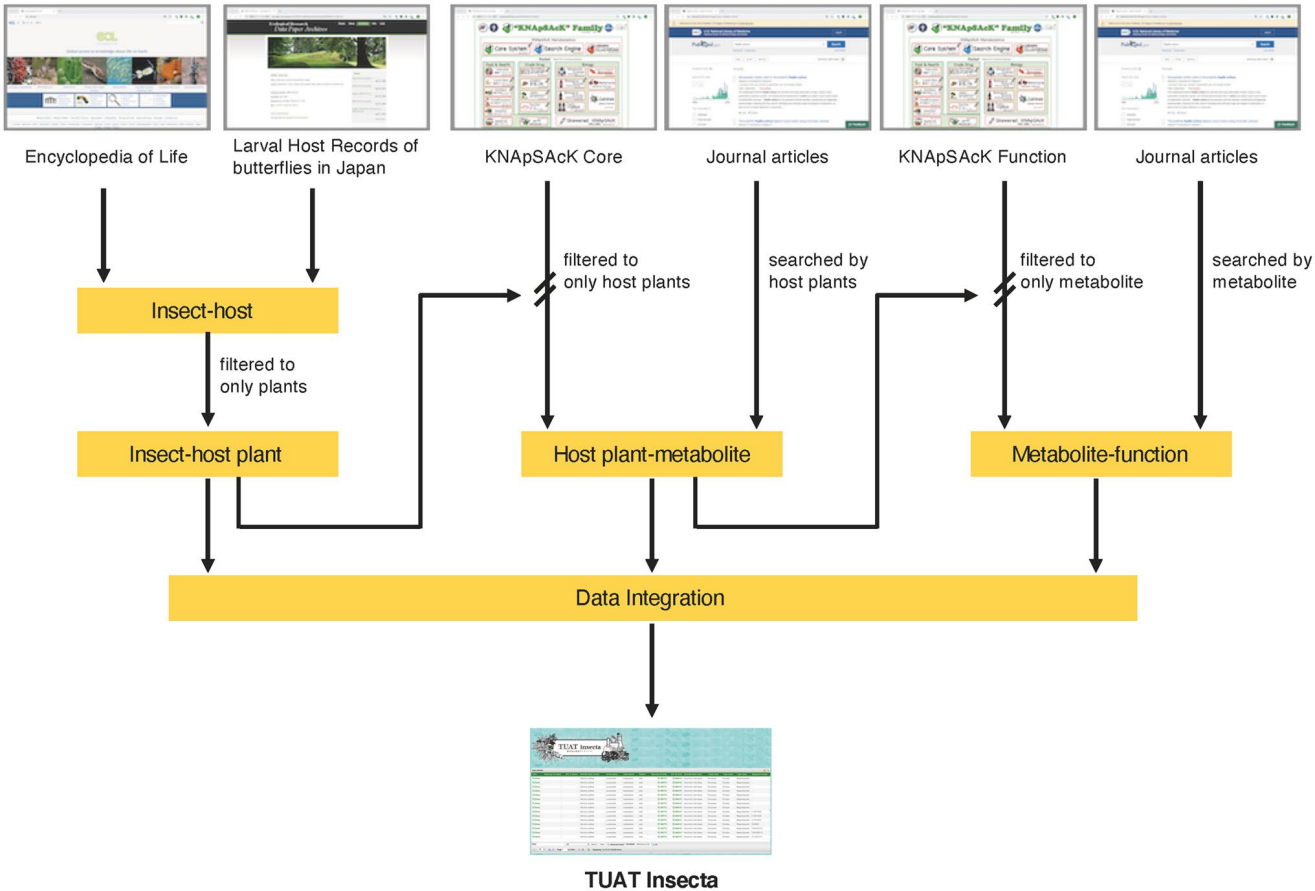
Outline for construction of the database TUAT insecta. Information about insect-host plant, host plant-metabolite and metabolite-function interactions were collected from public data sources and then integrated into a database, called TUAT insecta.

We collected information about insect-host plant, host plant-secondary metabolite and metabolite-biological function interactions from public data sources and then integrated these into a database (Fig. 1). As a result, our database TUATinsecta (https://togodb.org/db/tuat_insecta) contained 1,092,574 relationships between 15,505 insects and 11,578 host plants, and assigned 12,807 metabolites and 2,857 biological functions to 2,831 plants. Overall, TUATinsecta provided 1,132,984 relationships among insects, host plants, metabolites in plants, and the biological activity of metabolites. Lepidopteran insects comprise 10,506 (67.8%) of the insects in TUATinsecta (Table 1). Also, the breakdown of the 11,578 registered plants species revealed that 88.5% were plants eaten by butterflies or moths: Fabales, 9.4%; Poales, 7.3%; Lamiales, 6.8%; Malpighiales, 6.6%; Rosales. 6.3%; Asterales, 5.3%; Sapindales, 4.4%; and Gentianales, 4.2% (Table 2).

**Table 1:** Number of entries of insects to TUATinsecta.

Order	Number of entries
Lepidoptera	10506
Coleoptera	1162
Hemiptera	1029
Diptera	775
Hymenoptera	308
Trichoptera	98
Other	314

**Table 2:** Number of entries of plants to TUAT insecta (plants = 64 orders).

Order	Number of entries
Fabales	1088
Poales	846
Lamiales	791
Malpighiales	763
Asterales	608
Rosales	732
Sapindales	510
Gentianales	490
Caryophyllales	320
Myrtales	249
Brassicales	377
Malvales	334
Ericales	313
Apiales	306
Fagales	214
Asparagales	156
Arecales	190
Solanales	183
Saxifragales	140
Ranunculales	129
Other	1460

Next, using TUATinsecta, we investigated the biological activity of each plant fed on Lepidopteran insects; among the available studies, we encountered reports on diabetes-related (0.18%), cardiotonic (0.85%), antioxidant (1.5%), anti-inflammatory (2.3%), and anticancer activity (2.4%) in these plants, which had already had been used historically as folk medicine or herbal medicine. We were interested in whether or not the insect metabolic system changes second metabolites from these plants. Information in the database suggested that these plant second metabolites could change through the metabolic system in the insect. Some of the uses of Lepidopteran insects for secondary metabolites of plants included changing part of their chemical structure to become pheromones, kairomones, or defensive substances^15–18^. To compare the biological activity among the varieties of host plants metabolised by insect, we chose the Asian swallowtail *P. xuthus*, which eats several species of Rutaceae plants.

### Comparison of substances in different plants through swallowtail metabolic processes

**Figure 2:**
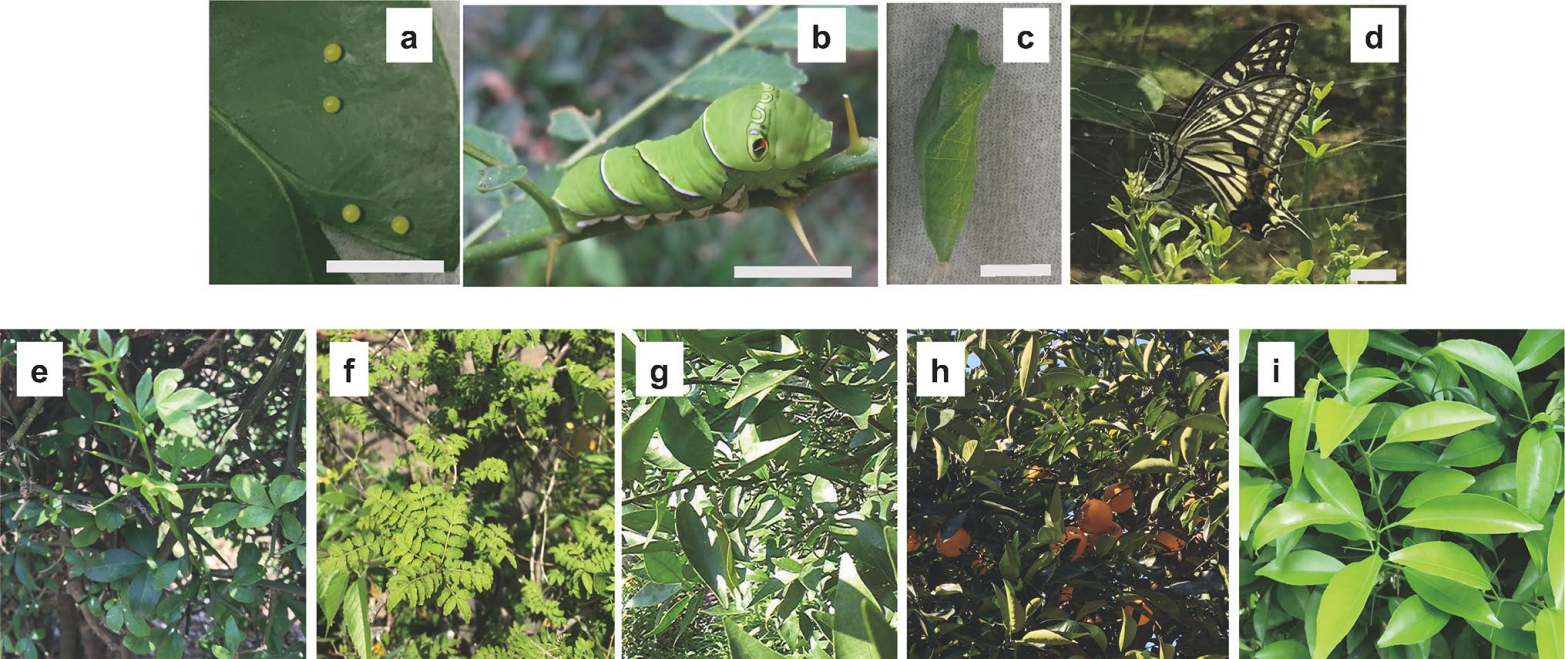
Plants and insects used in this study. (**a**–**d**) Developmental stages of *P. xuthus*. (**a**) *P. xuthus* egg, (**b**) *P. xuthus* final instar larva, (**c**) *P. xuthus* pupa, and (**d**) *P. xuthus* adult. The bars indicate 1 cm. (**e**–**i**) The five kinds of plants that are the host plants for larvae of *P. xuthus*. (**e**) *P. trifoliata*, (**f**) *Z. piperitum*, (**g**) *C. sudachi*, (**h**) *C. natsudaidai*, and (**i**) *C. junos.*

The Asian swallowtail, *P. xuthus* is Lepidopteran insect. Their life stages are shown as Fig. 2a–d. They feed on several species of Rutaceae plants during the larval developmental stage (Fig. 2b), and we examined whether secondary metabolic products including these plants may be altered further through the metabolic process in *P. xuthus*. Using three types of organic solvents of differing polarity, we extracted the metabolic products from each frass of *P. xuthus* larvae which fed on *Poncirus trifoliata* (Fig. 2e), *Zanthoxylum piperitum* (Fig. 2f), *Citrus sudachi* (Fig. 2g), *Citrus natsudaidai* (Fig. 2h), or *Citrus junos* (Fig. 2i). In Supplementary Table 1 we show the dry weight and extraction weight in each sample. The extraction dry weight of all frass methanol extracts (MeOH ext.) were higher than those of *n*-hexane extracts (Hex ext.) and chloroform extracts (CHCl_3_ ext.) in this study.

**Figure 3:**
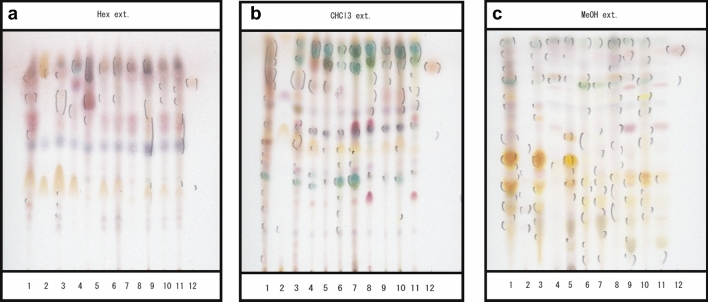
Examination of the substances from each extraction separated by TLC. We separated each substance with chloroform:methanol, 20:1 (v/v) for *n*-hexane ext., and 10:1 (v/v) for CHCl_3_ ext., chloroform:methanol:distilled water = 6: 4: 1 (v/v) for MeOH ext. as the developing solvents on TLC. (**a**) hexane extract from each sample, (**b**) chloroform extract, (**c**) methanol extract. 1, frass from *P. trifoliata*; 2, pupa from *P. trifoliata*; 3, leaves from *P. trifoliata*; 4, frass from *Z. piperitum*; 5, leaves from *Z. piperitum*; 6, frass from *C. sudachi*; 7, leaves from *C. sudachi*; 8, frass from *C. natsudaidai*; 9, leaves from *C. natsudaidai*; 10, frass from *C. junos*; 11, leaves from *C. junos*; 12, Auraptene.

We compared each metabolic product between leaf and frass in MeOH ext., Hex ext., or CHCl_3_ ext., by thin layer chromatography (TLC). Consequentially, we found several spots in the frass extracts which not evident in each corresponding plant extract (Fig. 3a–c, lane 1, 4, 6, 8, 10).

### Observation of the morphological changes to human cancer cell lines

**Figure 4:**
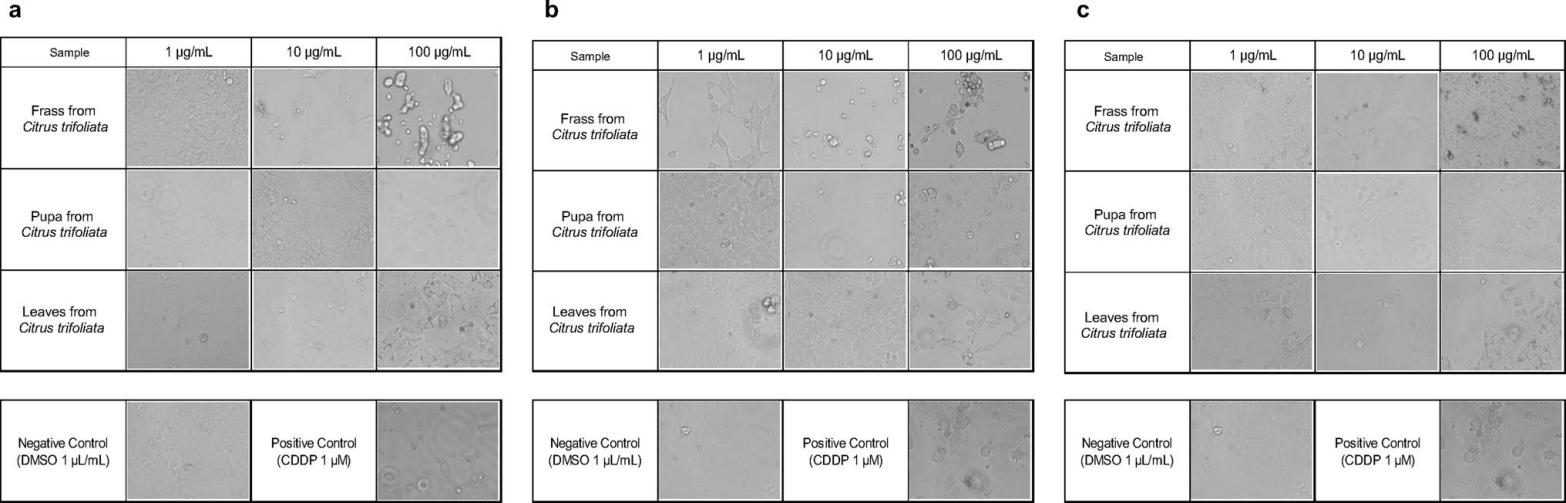
Observation of the morphological change to each human cancer cell line after treatment with each extraction. (**a**) HepG2 cell line; (**b**) HeLa cell line; (**c**) MIA-Paca2 cell line.

We evaluated the cytotoxic activity of the substances extracted from each sample on three human cancer cell lines: the human liver cancer cell line (HepG2), the human uterine cancer cell line (HeLa), and the human pancreatic cancer cell line (MIA Paca-2). First, we examined morphological changes using the phenotypic change score in these human cancer cell lines (Supplementary Table 2, a–g). We found that the frass from *P. trifoliata* CHCl_3_ ext. induced cell death in all three human cancer cell lines (Supplementary Table 2, a, c, e). We observed the strongest phenotypic change based on the phenotypic change score in the frass from *P. trifoliata* CHCl_3_ ext. on HepG2 and MIA-Paca2 (Fig. 4a, c). The frass from *P. trifoliata* CHCl_3_ ext. suppressed HepG2, HeLa, and MIA Paca-2 cell growth and cell viability, or induced morphological changes as assessed by the phenotypic change score. The CHCl_3_ ext. from pupae which had been fed on *P. trifoliata* had no effect on HepG2, HeLa, or MIA-Paca2 cells (Fig. 4, Supplementary Table 2-g).

### Evaluation of each extract on cell survival

**Figure 5:**
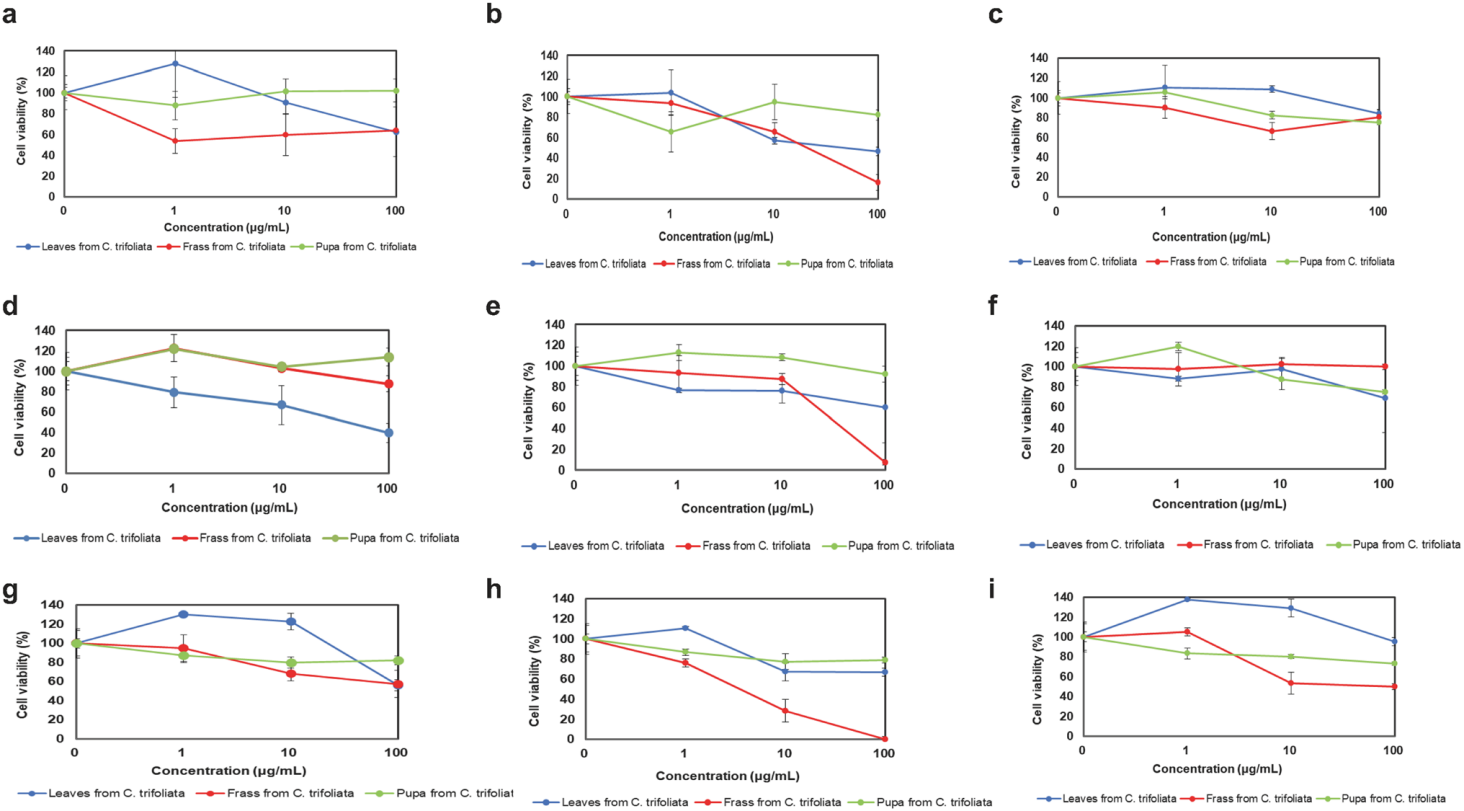
Evaluation of cell viability by MTT assay in the extracts of leaves from *P. trifoliata*. We added the extract to each cell, and then evaluated cell viability by MTT assay. (**a**) HepG2 treated with hexane extracts, (**b**) HepG2 treated with chloroform extracts, (**c**) HepG2 treated with methanol extracts, (**d**) HeLa treated with hexane extracts, (**e**) HeLa treated with chloroform extracts, (**f**) HeLa treated with methanol extracts, (**g**) MIA-Paca2 treated with hexane extracts, (**h**) MIA-Paca2 treated with chloroform extracts, (**i**) MIA-Paca2 treated with methanol extracts.

We next evaluated cell survival capability of extracts from *P. trifoliata* frass and leaf extracts and insect pupa which had been fed on *P. trifoliata* leaves. HepG2, HeLa, and MIA Paca-2 cells were treated with each extract and cell viability of each cell line was evaluated by MTT assay (Fig. 5, Supplementary Table 3, a–c). In Supplementary Table 3, a–c, we show the calculated estimated IC_50_ of each extract. The frass CHCl_3_ ext. derived from *P. trifoliata* induced dose-dependent cell death in HepG2, HeLa, and MIA Paca-2 (Fig. 5). We calculated estimated IC_50_ of frass CHCl_3_ ext. derived from *P. trifoliata* to be 11.8 μg/mL for HepG2 cells, 51.8 μg/mL for HeLa cells, and 3.7 μg/mL for MIA Paca-2 cells, and the IC_50_ of leaf CHCl_3_ ext. derived from *P. trifoliata* was 11.7 μg/mL in HepG2 cells, and 100 μg/mL or more in HeLa cells and MIA Paca-2 cells. These data clearly demonstrate that frass CHCl_3_ ext. derived from *P. trifoliata* induced storong cell death in MIA Paca-2 and HepG2 cell lines more strongly than leaf CHCl_3_ ext. (*p* = 0.006 for MIA Paca-2, *p* = 0.004 for HepG2). No statistical difference was found in the frass CHCl_3_ ext. derived from *P. trifoliata* in the HeLa cell line (*p* = 0.054) compared with that in the leaf CHCl_3_ ext. Furthermore, frass CHCl3 extract derived from *C. junos* also induced cell death in HepG2 (*p* = 0.0004), HeLa (*p* = 0.0004), and MIA Paca-2 (*p* = 0.025) cell lines to a greater extent than in the leaf CHCl_3_ extract (Supplementary Fig. 1 and Supplementary Tables 2b and 2c). Frass CHCl3 extract derived from *C. sudachi* induced cell death in HepG2 (*p* = 0.019), and MIA Paca-2 (*p* = 0.0006) cell lines than the leaf CHCl3 extract derived from *C. sudachi* (Supplementary Fig. 2 and Supplementary Tables 3a and 3c). Extractions from larval frass which had been fed on different types of plant leaf differed in their ability to induce cell death.

### Comparison of the metabolic products from *P. trifoliata* frass and leaves

The frass CHCl_3_ ext. derived from *P. trifoliata* induced cell death to human cancer cells more strongly than that from other plants frass CHCl_3_ exts. Inspection the TLC plate chromatogram at long wavelength UV irradiation (365 nm) revealed seven spots with calculated relative to front (R_f_) values of 0.1 to 0.3 and 0.5 to 0.7 in the frass of *P. trifoliata* CHCl_3_ ext. (Supplementary Fig. 3-b lane 1, red open arrows). We did not find these spots in the leaf of *P. trifoliata* CHCl_3_ ext. (Supplementary Fig. 3-b lane 3). We also detected a green-coloured spot with an R_f_ value of 0.4 in *P. trifoliata* frass CHCl_3_ ext. (Supplementary Fig. 3-b lane 1, closed red arrows), and a similar green-coloured spot with an R_f_ value of 0.3 in *P. trifoliata* CHCl_3_ ext. (Supplementary Fig. 3-b lane 3, closed red arrows), and there are two distinct spots with an R_f_ value of 0.8 to 0.9 in the CHCl_3_ extract derived from leaves of *P. trifoliata* not found in the CHCl_3_ extract derived from frass of *P. trifoliata* [Supplementary Fig. 3, both green colored spots are shown with closed black arrows (right side)]. Furthermore, we detected a spot by short wavelength irradiation (254 nm) of ultraviolet irradiation with an R_f_ value of 0.6 in *P. trifoliata* leaf CHCl_3_ ext. (Supplementary Fig. 3-b lane 3 open red arrows); however, we did not detect such a spot in *P. trifoliata* frass CHCl_3_ ext.

**Figure 6:**
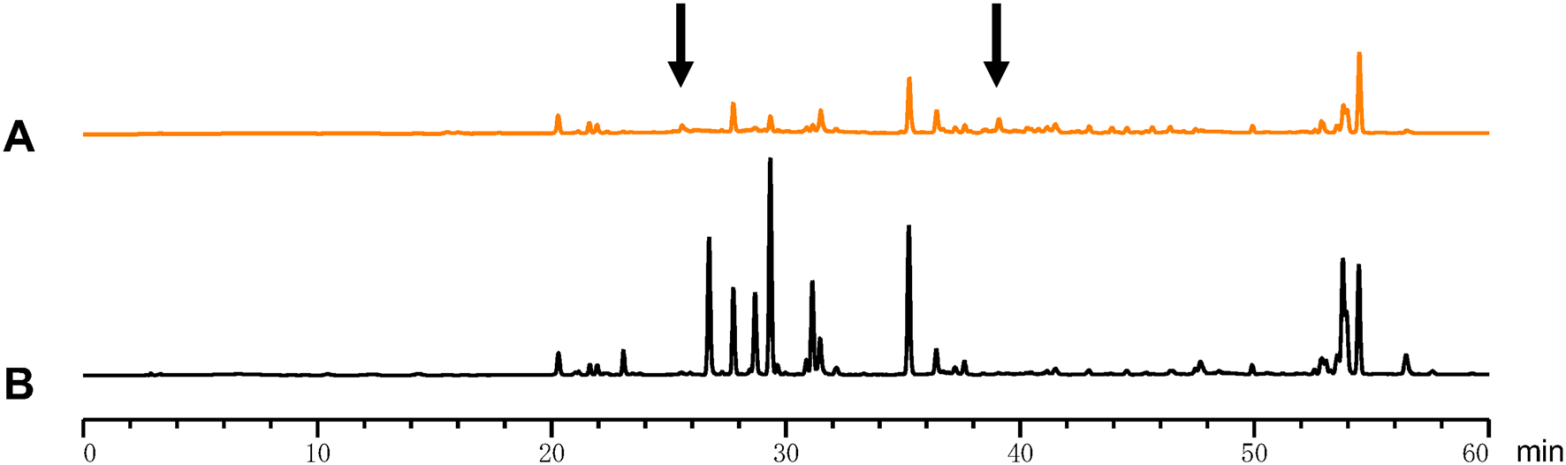
Comparison of substances from leaves with those from frass extract in *P. trifoliata.* We compared CHCl_3_ extracts from leaves of *P. trifoliata* and frass of *P. trifoliata* using HPLC.

Taken together, TLC analysis indicates that the composition of the metabolic products in *P. trifoliata* frass CHCl_3_ ext. differed from those in the *P. trifoliata* leaf CHCl_3_ ext.. Comparison of *P. trifoliata* frass CHCl_3_ ext. and *C. trifoliata* leaf CHCl_3_ ext. by high performance liquid chromatography (HPLC) revealed two peaks (Fig. 6a, arrows). These peaks were not observed in *P. trifoliata* leaf CHCl_3_ ext. (Fig. 6b). The peaks of the retention times were 26 min and 39 min (Fig. 6a, arrows).

## Discussion

Here, we constructed “TUATinsecta” by data integration to efficiently find the phytophagous insect species to screen for pharmaceutical resources and to experimentally determine whether the metabolic products from the host plants would be changed by insect metabolic system.

First, using several insect databases^19,20^, we entered the information regarding insect species which fed on plants, thereafter adding the information from the KNApSAcK database^21^ about their host-plant substances and their biological activity; we then created connections among the various information fields in the TUATinsecta database.

We found 2857 insect–host plants relationships with some biological activity on the organisms. We investigated the information on biological activity for each host plant, and discovered reports of anticancer activity for many of them. We also found reports on diabetes, cardiotonic, antioxidant, anti-inflammatory and antibacterial effects in many host plants based on past records of folk medicines and herbal medicine.

Phytophagous insects take up metabolic products from their host plants during the larval developmental stage. These substances are mainly hydrocarbons, proteins and lipids, which are categorised as primary metabolites^22^. At the same time, phytophagous insects must ingest several types of secondary metabolites—such as alkaloids, terpenoids, flavonoids, polyketides or phenylpropanoids—from their host plants when they feed on them^23^. These secondary metabolites may have affect larval growth, development and eating behaviour^24,25^.

The presence of enterobacteria has been confirmed in *Papilio polytes*, but most are from plants. These bacteria do not seem to be involved in the metabolic process in *Papilio polytes*^26^. Furthermore, the administration of antibiotics to larvae of *Danaus chrysippus* and *Ariadne merione* does not affect their growth and survival^27^. Thus, we speculated that the enterobacteria are not involved in the metabolism of secondary plant metabolites in Lepidopteran insects. As a further support, the pupal extract does not affect the viability of human cancer cells. Accordingly, we considered that the larva must have a specific metabolic system when feeding on host plants for avoiding the effects of these secondary metabolites on their growth, development and eating behaviour. Presumably, host plant secondary metabolites are processed in the larval body through their special metabolic system and appear in an altered form in frass.

Cytochrome P450 (CYP) plays a role for detoxication of these secondary metabolites in phytophagous insects and has been suggested to relate to the host plant specialisation in phytophagous insects^28^. In the detoxication process, specific plant secondary metabolites are metabolised by CYPs, and the final metabolites are then processed to frass. Both the insect body and the larval frass may also contain metabolised substances not included in the original plant substances. For this reason, we anticipated that frass would contain biologically active agents because the secondary metabolites from the host plant could be changed through the insects’ metabolic system. From this standpoint, we examined the biological activity of five kinds of frass obtained from the Asian swallowtail *P. xuthus*, which feeds on *P. trifoliata*, *Z. piperitum*, *C. natsudaidai*, *C. junos* or *C. sudachi*.

In comparing the biological activity of each extract, we found that CHCl_3_ ext. *P. trifoliata* frass induced strong cell death and morphological change in the HepG2, HeLa and MIA-Paca2 cell lines; CHCl_3_ ext. from *C. junos* frass, however, induced strong cell death and morphological change only in the HeLa cell line. In contrast, CHCl_3_ ext. from the frass of *Z. piperitum*, *C. sudachi*, and *C. natsudaidai* did not induce strong cell death and morphological change to the HepG2, HeLa or MIA-Paca2 cell lines. We speculated plants secondary metabolite from *P. trifoliata* or *C. junos* might be altered the chemical structure by the metabolic system in *P. xuthus*.

These altered substances included in the CHCl_3_ fraction could induce cell death. CHCl_3_ is a less polar solvent. We thus considered these substances potentially to be lipophilic compounds of low molecular weight.

Plants in the Rutaceae include flavonoids, coumarins, carotenoids and limonoids. Some of these substances have effects on the human cancer cell line (Supplementary Table 4)^29,30^. The coumarin derivative, auraptene found in the *C. natsudaidai* was reported to show anticancer activity^31^. We were curious as to whether the auraptene was included in the frass or the leaf extract of *P. trifoliata*; HPLC indicated an auraptene peak at 44 min at RT in the Hex ext. of frass and leaf of *P. trifoliata* (Supplementary Fig. 4a and b). However, neither of the Hex exts. induced cell death in the HepG2, HeLa or MIA-Paca2 cell lines (Supplementary Table 2, and Fig. 5a,d,g). Therefore, we hypothesised that a compound other than auraptene was altered by the *P. xuthus* metabolic system to induce cell death. According to TUATinsecta, *P. trifoliata* includes several known biologically active substances such as xanthotoxol and imperatorin. These compounds lead to proliferation in the HeLa cell line. In comparing *P. trifoliata* frass and leaf CHCl_3_ ext. by HPLC, we identified two specific peaks at 26 min and 39 min for CHCl_3_ ext. from *P. trifoliata* frass using HPLC (Fig. 6). However, we could not identify chemical property on these specific peaks. We predict that a very small amount of the substance or complex of several substances in the frass of *P. trifoliata* can induce cell death.

Some insect frass have a history of use as a folk medicine. In China, San-sha—the dried frass obtained from young silkworm larvae, a known as Chinese herbal medicine—has been used against inflammatory diseases such as rheumatism, joint pain, neuralgia, uterine bleeding, irregular menstruation, and conjunctivitis^32^. In addition, China has a tea called Chongshicha, made using the dried frass obtained from several kinds larvae of Pyralidae or Noctuidae^33^. Malaysia also has a tea made using dried frass obtained from stick insects that feed on guava leaves^34^. Some Malaysians believe this tea is effective against asthma, gastrointestinal disorders, and muscle pain^35^. Thus, Asian countries have a tradition of drinking tea made from frass of phytophagous insects by decoction. Silkworms eats mulberry leaves and stick insects eat guava leaves, and these plants include biologically active substances^36–39^. In this way, insect frass has long been used as folk medicine; however, the plant substances exhibiting pharmacological actions have, to date, not been clearly identified. Thus, if the plant contains secondary metabolic products that exhibit biological activity, it could be changed by insect metabolism into the metabolic products with even stronger biological activity. In this study, we integrated information on insect-host plants, host plant-secondary metabolites, and metabolite-biological function into TUATinsecta. TUATinsecta will assist investigators to find specific compounds, including plant-derived secondary metabolic products, in specific plants. Therefore, TUATinsecta is a helpful resource for choosing phytophagous insect species which could prove useful in identifying pharmaceutical candidates beneficial to humans.

In conclusion, we constructed a database for choosing phytophagous insect species which could convert plant-derived secondary metabolites through the insect metabolic system to metabolic products with biological activity. With the aid of TUATinsecta we chose candidate insect species and identified *P. xuthus*, which feeds on several species of Rutaceae plants. Using three types of organic solvents, we extracted the metabolic products from the frass produced by the metabolic system of *P. xuthus* after feeding on each Rutaceae plant. We evaluated the biological activity of these extracts by cell death to the human cancer cell line, and found that *P. xuthus* produced a special substances included in the frass when *P. xuthus* fed on *P. trifoliata*. Our results demonstrate that Lepidopteran insects can alter plant metabolic products, creating new substances with different biological activity. However, identification of these biologically active substances must await development of large-scale insect frass collecting methodologies.

In future studies, we will elucidate the biologically active substance included in the frass when *P. xuthus* fed on *P. trifoliata*, and try to convert the biological activity of substances by *P. xuthus* metabolic enzymes.

Furthermore, we intend to construct a technology safe to elaborate to artificially alter the chemical structure of plant-derived secondary metabolic products by insect metabolic enzymes.

Collectively, the results of our study will contribute to the identification of phytophagous insect species that have the ability to change plant substances into potential therapeutic agents via the insect metabolic system.

## Methods

### Construction of the dataset for TUATinsecta

To determine the relationships among insects, plants and their substances, we developed a database connecting the information among these fields of study. First, we obtained information for host plants of insects by downloading the Encyclopedia of Life database (https://eol.org/)^19^ for the “eats” attribute as of August 2019. We added further information for host plants to the retrieved data from the database^20^. We also searched field guides and extracted host record information. We then obtained information for substances included in all insects-host plants from the KNApSAcK database (https://www.knapsackfamily.com)^21^, which contains species-metabolite relations for plants and the biological activities and functions of these metabolites. We also extracted descriptions of biological functions from PubMed (https://www.ncbi.nlm.nih.gov/pubmed). Finally, we integrated this information-including insect species, insect–host plants, plant substances, and their biological activity-using a tab-delimited formatted file. We also established hyperlinks to the corresponding web pages in the NCBI Taxonomy to facilitate the access to this information from TUATinsecta using the scientific names of insects and plants. We used TogoDB platform (Database hosting service, https://togodb.org/) for the visualization of the tab-delimited file. We then published the dataset as TUATinsecta on the platform. Imported data are converted to an RDF-ized format. TUATinsecta is freely available at https://togodb.org/db/tuat_insecta under Creative Commons Attribution-ShareAlike 4.0 International (CC BY-SA 4.0) license (https://creativecommons.org/licenses/by-sa/4.0/). All users can search targeted information with specific keywords derived from insect, insect-host plants, substances or biological activity. Users can also download these data in CSV, JSON and RDF format. We will update TUATinsecta when each public database used for the construction of TUATinsecta is updated.

### Insect

We collected *Papilio xuthus* larvae from Fuchu City, Tokyo, Japan and Tokushima City, Tokushima Prefecture, Japan. A landowner permitted us to pick swallowtail butterfly *Papilio xuthus*. We maintained the Fuchu *P. xuthus* larvae on *Poncirus trifoliata*, *Zanthoxylum piperitum*, *Citrus natsudaidai* and *Citrus junos*. We harvested these plants at Fuchu campus, Tokyo University of Agriculture and Technology, Fuchu city, Tokyo, Japan. We maintained the Tokushima *P. xuthus* larvae on *Citrus sudachi*. We harvested *Citrus sudachi* at Tokushima city, Tokushima Prefecture, Japan. We maintained *P. xuthus* larvae at 25 °C with a 16-h/8-h light/dark cycle, and mated the emerged adult individuals to collect eggs, which we reared under the same conditions from larval to pupal developmental stage after hatching. We collected *P. xuthus* frass during the larval developmental stage in the course of maintenance with each plant. We used *P. xuthus* pupae maintained with *P. trifoliata* during the larval developmental stage. All samples stored at − 30 °C until extraction of substances.

### Extraction of substances from plants and several kinds of *P. xuthus* frass

We placed each sample on a glass petri dish and freeze-dried it with lyophilizer (VD-250F, TAITECH Co., Ltd., Saitama, Japan) for 24 h. We pulverized each sample and weighed it using a Mettler electronic scale. We transferred the weighed sample to a 300 mL Erlenmeyer flask, and added 3 volumes of *n*-hexane (Hex; Wako Pure Chemical Industries, Ltd., Osaka, Japan). We left the mixture for 72 h at RT and then filtered it. We transferred the pellet to a 300 mL Erlenmeyer flask, added 3 volumes of Hex, left it for 72 h at RT, and then filtered it. We repeated this operation one more time, and transferred the filtrate to an eggplant flask for evaporation.

We transferred the pellet to a 300 mL Erlenmeyer flask, and added 3 volumes of chloroform (CHCl_3_; Wako Pure Chemical Industries, Ltd.) to the pellet. We then conducted the same operation with Hex.

We then transferred the pellet to a 300 mL Erlenmeyer flask and added 3 volumes of methanol (MeOH; Wako Pure Chemical Industries, Ltd.) to the pellet. We then conducted the same operation with Hex.

We concentrated these filtrates, including *n*-hexane, CHCl_3_ or MeOH, by rotary evaporator (N-1110N, Tokyo Scientific Instruments Co., Ltd., Tokyo, Japan). Finally, we collected each extract in a screw tube (No. 7, Marem Co., Ltd., Osaka, Japan) and kept them in a vacuum desiccator for 3–5 days to completely remove each organic solvent, and then weighed the obtained extract. We shielded the screw tubes containing the extracts from light with aluminium foil and stored them at 4 °C until use. We referred to the extract with *n*-hexane as Hex ext., the extract with CHCl_3_, as CHCl_3_ ext., and the extract with MeOH, as MeOH ext.

### Comparison of components of extracts by thin layer chromatography (TLC)

For each extract, we dissolved samples in appropriate organic solvents and spotted 1–2 μL on a TLC plate (silica gel 60 F 254, 8 cm long) (Merck Millipore, CA, USA) using a glass capillary. We immersed the bottom of the TLC plates in an organic solvent until the solvent front reached approximately 7 cm from the origin. We chose chloroform:methanol, 20:1 (v/v) for *n*-hexane ext., chloroform:methanol 10:1 (v/v) for CHCl_3_ ext. and, chloroform:methanol:distilled water = 6: 4: 1 (v/v) for MeOH ext. as the developing solvents on TLC. For visualising each spot indicating an substance, we excited the spots by ultraviolet light (short wavelength; 254 nm, long wavelength; 365 nm) using a UV irradiator (UVGL-15, Funakoshi Co., Ltd., Tokyo, Japan). Thereafter, we also sprayed the TLC plate with 50% sulfuric acid (Wako Pure Chemical Industries, Ltd., Osaka, Japan) and heat-treated it at 120 °C for 2 min after the extract had separated. We recorded the position of each spot relative to the solvent front (Rf). Rf value was calculated as the distance from baseline traveled by the solute divided by the distance from baseline traveled by the solvent (solvent front).

### Preparation of chemicals and evaluation of cell viability

We dissolved each extract in dimethyl sulfoxide (DMSO; Sigma-Aldrich, MO, USA) to a concentration of 1 mg/mL, 10 mg/mL, and 100 mg/mL to prepare an extract solution. Each extract solution was protected from light by shading.

We used cisplatin (CDDP; Wako Pure Chemical Industries, Ltd., Osaka, Japan) as a positive control (PC). We dissolved CDDP in phosphate buffered saline (PBS; 8.1 mM NaHPO4, 137 mM NaCl, 2.7 mM KCl, 1.47 mM KH2PO4, pH 7.4), adjusted to 1 mM, and protected it from light by shading. We used an equivalent concentration (v/v) of DMSO as negative controls.

We obtained the human liver cancer cell line HepG2 (RCB1886), the human cervical cancer cell line HeLa (RCB0007), and the human pancreatic cancer cell line MIA Paca-2 (RCB2094) from RIKEN cell bank (RIKEN Tsukuba, Japan). We cultured each cell line in Dulbecco's modified Eagle's medium (Wako Pure Chemical Industries, Osaka, Japan) supplemented with 10% (v/v) fetal bovine serum (Sigma-Aldrich, MO, USA), 100 U/ml penicillin, and 100 μg/ml streptomycin solution (Wako Pure Chemical Industries, Ltd., Osaka, Japan), at 37 °C under 5% CO_2_. We cultured cell lines until sufficient cell stock obtained and then stored cells at − 80 °C until use. The second passage number of cells was used in all experiments. To assess the effect of the extract on each cell line, we examined cell viability by MTT assay (Merck Millipore, CA, USA).

We seeded each cell line at 2 × 10^3^ cells/50 μL/well on a 96-well plate (Nippon Becton Dickson Co., Ltd., Tokyo, Japan), and then cultured them for 24 h at 37 °C under 5% CO_2_. After 24 h, we treated each well with the extract solution at final concentrations of 1 μg/mL, 10 μg/mL, and 100 μg/mL (0.1% (v/v) DMSO), respectively, and continued the culture for a further 72 h. We used cells treated with DMSO at a concentration of 0.1% (v/v) as a negative control. We added 10 μL of MTT reagent to each well of the 96 well plate after culture for 72 h, and performed the culture for a further 3 h. Thereafter, we added 100 μL of a solution of 0.04 N hydrochloric acid–isopropanol solution to each well to dissolve the formazan dye included in the cells, and then measured the absorbance at 540 nm and 620 nm using a plate reader (iMark, Bio Rad, The absorbance CA, USA). We calculated the cell viability with the following equation:

$$ {\text{cell}}\;{\text{viability}}\;(\% ) = ([{\text{sample}}\;{\text{absorbance}}] - [{\text{blank}}\;{\text{absorbance}}])/([{\text{control}}\;{\text{absorbance}}] - [{\text{blank}}\;{\text{absorbance}}]) \times 100 $$

### Observation of cell morphology

We observed the cell morphology of the extract on each cell line.

We seeded each cell line at 0.5 × 10 ^5^ cells/500 μL/well on a 24-well plate (IWAKI, Shizuoka, Japan) and cultured them for 24 h at 37 °C. under 5% CO_2_. After 24 h, we treated each well with the extract solution at final concentrations of 1 μg/mL, 10 μg/mL, and 100 μg/mL (0.1% (v/v) DMSO), respectively, and continued the culture for a further 72 h. We used CDDP at 1 mM as a PC. After 72 h, we observed the cell morphology using a stereo microscope (Floid Cell Imaging Station, Thermo Fisher). We categorised the cell morphology into five levels and scored it as follows: score 1: the cell proliferation and density are similar to those of the negative control group; score 2: the levels of cell proliferation and density are between those for score 1 and those for score 3; score 3: the cell density has decreased without cell morphological change; score 4: the cell density has decreased to between that for score 3 and that for score 5 by cell death; score 5: the cell density has significantly decreased, similar to that for the PC group, by cell death.

### Comparison of both extracts by high performance liquid chromatography

We used CHCl_3_ extracts from *P. trifoliata* leaf and frass, respectively. We adjusted the concentration of each extract to 10 µg/mL with acetonitrile (Sigma-Aldrich co. ltd., MO, USA) and then filtrated them using a 0.2 mm x φ 4 mm filter (AS ONE co. ltd. Osaka, Japan). We collected each filtrate in a 300 μL vial (Waters co. ltd. Tokyo, Japan).

We loaded these samples to a HPLC system comprising a JASCO PU-2089 (JASCO co. ltd. Tokyo, Japan), a JASCO UV-2075 (JASCO co. ltd. Tokyo, Japan), and a RHEODYNE 7125 (Rheodyne co. ltd, CA, USA) with a CAPCELL PAK C18 MG II column (4.6 φ × 250 mm)(SHISEIDO co. ltd., Tokyo Japan), pre-equilibrated with 20% acetonitrile. We set the wavelength to 220 nm, and the mobile phase was H_2_O: acetonitrile = 80:20 (0–10 min), 80:20 → 0:100 (10–50 min), and 0:100 (50–60 min) at a flow rate of 1.0 mL/min for 60 min. Separation on HPLC was conducted at room temperature.

### Statistical analysis

Statistical significance was determined by a two-tailed Student’s t-test using Excel (Microsoft, Redmond, WA, USA); *p *values of < 0.05 were significant.

## Supplementary information

Supplementary Table 1.

Supplementary Table 2.

Supplementary Table 3.

Supplementary Table 4.

Supplementary Figure 1.

Supplementary Figure 2.

Supplementary Figure 3.

Supplementary Figure 4.

## Data Availability

TUAT insecta is freely available at https://togodb.org/db/tuat_insecta under Creative Commons Attribution-ShareAlike 4.0 International (CC BY-SA 4.0) license (https://creativecommons.org/licenses/by-sa/4.0/).
