# Author Correction: Construction of TUATinsecta database that integrated plant and insect database for screening phytophagous insect metabolic products with medicinal potential

**DOI:** 10.1038/s41598-021-89260-x

**Published:** 2021-05-04

**Authors:** Wakana Nakane, Hisashi Nakamura, Takeru Nakazato, Natsuki Kaminaga, Miho Nakano, Takuma Sakamoto, Maaya Nishiko, Hidemasa Bono, Isao Ogiwara, Yoshikazu Kitano, Kikuo Iwabuchi, Kaoru Kinoshita, Richard J. Simpson, Hiroko Tabunoki

**Affiliations:** 1 Department of Science of Biological Production, Graduate School of Agriculture, Tokyo University of Agriculture and Technology, 3‑5‑8 Saiwai‑cho, Fuchu‑shi, Tokyo, 183‑8509 Japan; 2 Database Center for Life Science (DBCLS), Joint Support‑Center for Data Science Research, Research Organization of Information and Systems (ROIS), Mishima, Shizuoka 411‑8540 Japan; 3 Department of Pharmacognosy and Phytochemistry, Meiji Pharmaceutical University, 2‑522‑1 Noshio, Kiyose‑shi, Tokyo, 204‑858 Japan; 4 Department of United Graduate, School of Agricultural Science, Tokyo University of Agriculture and Technology, 3‑5‑8 Saiwai‑cho, Fuchu‑shi, Tokyo, 183‑8509 Japan; 5 Department of Applied Biological Science, Tokyo University of Agriculture and Technology, 3‑5‑8 Saiwai‑cho, Fuchu‑shi, Tokyo, 183‑8509 Japan; 6 Department of Biochemistry and Genetics, La Trobe Institute for Molecular Science (LIMS), La Trobe University, Melbourne, VIC 3086 Australia; 7 Institute of Global Innovation Research, Tokyo University of Agriculture and Technology, 3‑5‑8 Saiwai‑cho, Fuchu, Tokyo 183‑8509 Japan

Correction to: *Scientific Reports* 10.1038/s41598-020-74590-z, published online 15 October 2020

The Article contains error in the Result Section under subheading ‘Development of the database “TUATinsecta” for choosing an insect’.

“As a result, our database TUATinsecta (https://togodb.org/db/tuat_insecta) contained 1,092,574 relationships between 15,505 insects and 11,578 host plants, and assigned 12,807 metabolites and 2,857 biological functions to 2,831 plants.”

should read:

“As a result, our database TUATinsecta (http://togodb.org/db/tuat_insecta) contained 1,092,574 relationships between 15,505 insects and 11,578 host plants, and assigned 12,807 metabolites and 2,857 biological functions to 2,831 plants.”

