# Supplementary material for: Construction of TUATinsecta database that integrated plant and insect database for screening phytophagous insect metabolic products with medicinal potential
Source: Sci Rep. 2020 Oct 15;10:17509. doi: 10.1038/s41598-020-74590-z (PMC7566601; doi:10.1038/s41598-020-74590-z)
Supplement: Supplementary file 1 — Supplementary Table 1. [file 41598_2020_74590_MOESM1_ESM.docx]

**Construction of TUATinsecta database that integrated plant and insect database for screening phytophagous insect metabolic products with medicinal potential**

Wakana Nakane ^1¶^, Hisashi Nakamura ^1¶^, Takeru Nakazato^2¶^, Natsuki Kaminaga^3^ Miho Nakano^1^, Takuma Sakamoto^4^, Maaya Nishiko^4^, Hidemasa Bono^2^, Isao Ogiwara^1^, Yoshikazu Kitano^5^, Kikuo Iwabuchi^1^, Kaoru Kinoshita^3^, Richard J. Simpson^6,7^, and Hiroko Tabunoki^1, 7*^

^1^Department of Science of Biological Production, Graduate School of Agriculture, Tokyo University of Agriculture and Technology, 3-5-8 Saiwai-cho, Fuchu-shi, Tokyo, 183-8509, Japan

^2^Database Center for Life Science (DBCLS), Joint Support-Center for Data Science Research, Research Organization of Information and Systems (ROIS), Mishima, Shizuoka, 411-8540, Japan

^3^Department of Pharmacognosy and Phytochemistry, Meiji Pharmaceutical University, 2-522-1 Noshio, Kiyose-shi, Tokyo, 204-8588, Japan.

^4^Department of United Graduate School of Agricultural Science, Tokyo University of Agriculture and Technology, 3-5-8 Saiwai-cho, Fuchu-shi, Tokyo, 183-8509, Japan

^5^Department of Applied Biological Science, Tokyo University of Agriculture and Technology, 3-5-8 Saiwai-cho, Fuchu-shi, Tokyo, 183-8509, Japan.

^6^Department of Biochemistry and Genetics, La Trobe Institute for Molecular Science (LIMS), La Trobe University, Melbourne, Victoria, 3086, Australia.

^7^Institute of Global Innovation Research, Tokyo University of Agriculture and Technology, 3-5-8 Saiwai-cho, Fuchu, Tokyo, 183-8509, Japan.

¶Wakana Nakane, Hisashi Nakamura, and Takeru Nakazato contributed equally

*Corresponding author: Hiroko Tabunoki

Tel: +81-42-367-5613; Fax: +81-42-367-5613

E-mail: [h_tabuno@cc.tuat.ac.jp](mailto:h_tabuno@cc.tuat.ac.jp)

E-mail addresses

Wakana Nakane:wakanananan0608@gmail.com

Hisashi Nakamura: [joenakamura819@gmail.com](mailto:joenakamura819@gmail.com)

Takeru Nakazato: [nakazato@dbcls.rois.ac.jp](mailto:nakazato@dbcls.rois.ac.jp)

Natsuki Kaminaga: enzian3575@gmail.com

Miho Nakano: nmh.101117@gmail.com

Takuma Sakamoto: tsakamoto1988@gmail.com

Maaya Nishiko: N.maaya1222@gmail.com

Hidemasa Bono: [bono@dbcls.rois.ac.jp](mailto:bono@dbcls.rois.ac.jp)

Isao Ogiwara:ogiwara@cc.tuat.ac.jp

Yoshikazu Kitano: [kitayo@cc.tuat.ac.jp](mailto:kitayo@cc.tuat.ac.jp)

Kikuo Iwabuchi:kikkuo@cc.tuat.ac.jp

Kaoru Kinoshita:kaoru@my-pharm.ac.jp

Richard J. Simpson: richard.simpson@latrobe.edu.au

Hiroko Tabunoki: h_tabuno@cc.tuat.ac.jp

**Supplementary information**

**Supplementary Table**

**Supplementary Table 1. Dry weight and extraction weight in each sample.**

| Sample | Sample dry weight (mg) | *n*-Hexane extraction (mg) | Chloroform extraction (mg) | Methanol extraction (mg) |
| --- | --- | --- | --- | --- |
| frass from *Poncirus trifoliata* | 2040 | 150 | 430 | 3710 |
| pupa from *Poncirus trifoliata* | 1100 | 40 | 20 | 140 |
| leaf from *Poncirus trifoliata* | 14000 | 90 | 420 | 3350 |
| frass from *Zanthoxylum piperitum* | 46000 | 310 | 530 | 3110 |
| leaf from *Zanthoxylum piperitum* | 6000 | 230 | 80 | 800 |
| frass from *Citrus sudachi* | 13000 | 20 | 70 | 80 |
| leaf from *Citrus sudachi* | 4500 | 340 | 1060 | 860 |
| frass from *Citrus natsudaidai* | 43800 | 160 | 440 | 1140 |
| leaf from *Citrus natsudaidai* | 16000 | 80 | 100 | 2460 |
| frass from *Citrus junos* | 22500 | 150 | 430 | 3710 |
| leaf from *Citrus junos* | 10000 | 40 | 200 | 1040 |

**Supplementary Table 2. Observation of the morphological change to each human cancer cell line after treatment with each extraction.** (a) Frass extract to the HepG2 cell line; (b) leaf extract to the HepG2 cell line; (c) frass extract to the Hela cell line; (d) leaf extract to the Hela cell line; (e) frass extract to the MIA-Paca2 cell line; (f) leaf extract to the MIA-Paca2 cell line; (g) pupae extracts to the HepG2, Hela, and MIA-Paca2 cell lines. We categorized the cell morphology into five levels and scored them as follows: score 1: the cell proliferation and density are similar to those of the negative control group; score 2: the levels of cell proliferation and density are between those for score 1 and those for score 3; score 3: the cell density has decreased without cell morphological change; score 4: the cell density has decreased to a level between that for score 3 and that for score 5, by cell death; score 5: the cell density has significantly decreased, similar to that of the positive control group, by cell death.

**Supplementary Table 3. IC50 (μg/mL) of the human cancer cell line treatment with each extract.** (A) HePG2, (B) Hela, (C) MIA-Paca2.

**Supplementary Table 4. The substances included in *P. trifoliata*.**

**Supplementary figures**

**Supplementary Figure 1. Evaluation of cell viability by MTT assay in the extracts of leaves from *C. natsudaidai* or *C. junos* and frass from *C. natsudaidai* or *C. junos*.** We added the extract to each cell, and then evaluated cell viability by MTT assay. (a) HepG2 treated with *n*-hexane extract; (b) HepG2 treated with chloroform extract; (c) HepG2 treated with methanol extract; (d) Hela treated with *n*-hexane extract, (e) Hela treated with chloroform extract; (f) Hela treated with methanol extract; (g) MIA-Paca2 treated with *n*-hexane extract; (h) MIA-Paca2 treated with chloroform extract; (i) MIA-Paca2 treated with methanol extract.

**Supplementary Figure 2. Evaluation of cell viability by MTT assay in the leaf extracts from *Z. piperitum* or *C. sudachi* and from the frass extracts from *Z. piperitum* or *C. sudachi***. We added the extract to each cell, and then evaluated cell viability by MTT assay. (a) HepG2 treated with *n*-hexane extract; (b) HepG2 treated with chloroform extract; (c) HepG2 treated with methanol extract; (d) Hela treated with *n*-hexane extract, (e) Hela treated with chloroform extract; (f) Hela treated with methanol extract; (g) MIA-Paca2 treated with *n*-hexane extract; (h) MIA-Paca2 treated with chloroform extract ; (i) MIA-Paca2 treated with methanol extract.

**Supplementary Figure 3. Examination of the ingredients from each extraction separated by TLC.** Each ingredient separated with chloroform:methanol, 20:1 (v/v) for *n*-hexane ext., chloroform:methanol, 10:1 (v/v) for CHCl_3_ ext., and chloroform:methanol:distilled water = 6: 4: 1 (v/v) for MeOH ext. as the developing solvents on TLC. (a) Hexane extract from each sample; (b) chloroform extract; (c) methanol extract. 1: frass from *P. trifoliata*; 2: pupa from *P. trifoliata*; 3: leaf from *P. trifoliata*; 4: frass from *Z. piperitum*; 5: leaf from *Z. piperitum*; 6: frass from *C. sudachi*; 7: leaf from *C. sudachi*; 8: frass from *C. natsudaidai*; 9: leaf from *C. natsudaidai*; 10: frass from *C. junos*; 11: leaf from *C. junos*; 12“: auraptene.

Seven spots found by short wavelength (254nm) of ultraviolet irradiation are shown with red open arrows (left side), and the green colored spots are shown with closed red arrows (right side), The spots detected by long wavelengths (365nm) of ultraviolet irradiation are shown with open red arrows (right side). We recorded the position of each spot relative to the solvent front (Rf). Rf value was calculated as the distance from baseline traveled by the solute divided by the distance from baseline traveled by the solvent (solvent front).

**Supplementary Figure 4. Comparison of ingredients in leaf extract with those in frass extract in *P. trifoliata.* We compared** *n*-hexane extracts from leaves of *P. trifoliata* with those from frass of *P. trifoliata* using HPLC. a, *n*-hexane extract from leaves of *P. trifoliata;* b, *n*-hexane extract from frass which fed on leaves of *P. trifoliata;* c, *n*-hexane extract from pupa which fed on leaves of *P. trifoliata;* d, auraptene. A black arrow indicates the peak for auraptene.
