# Supplementary figures and images for: Construction of TUATinsecta database that integrated plant and insect database for screening phytophagous insect metabolic products with medicinal potential
Source: Sci Rep. 2020 Oct 15;10:17509. doi: 10.1038/s41598-020-74590-z (PMC7566601; doi:10.1038/s41598-020-74590-z)

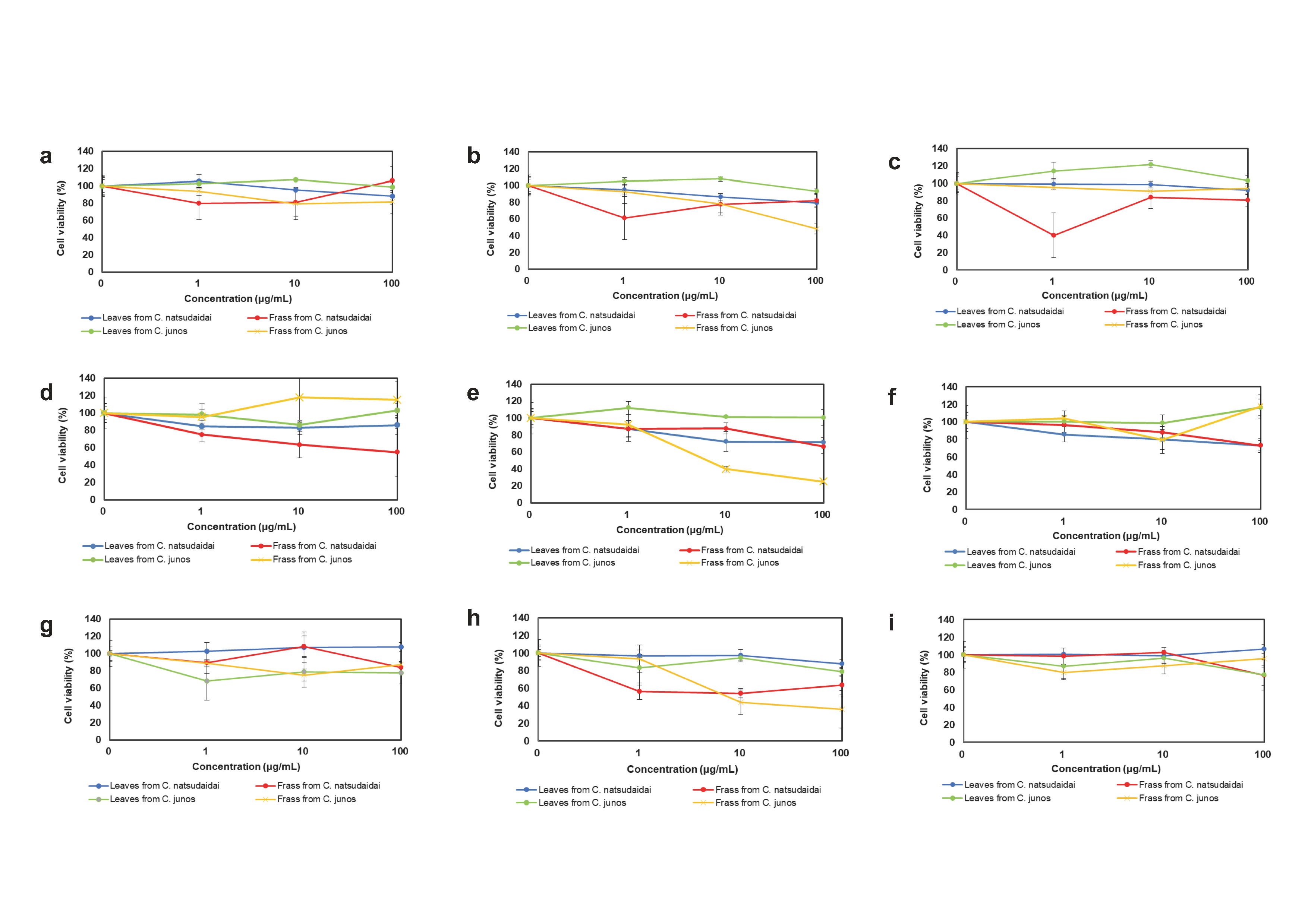

Supplement: Supplementary file 5 — Supplementary Figure 1. [file 41598_2020_74590_MOESM5_ESM.tiff]

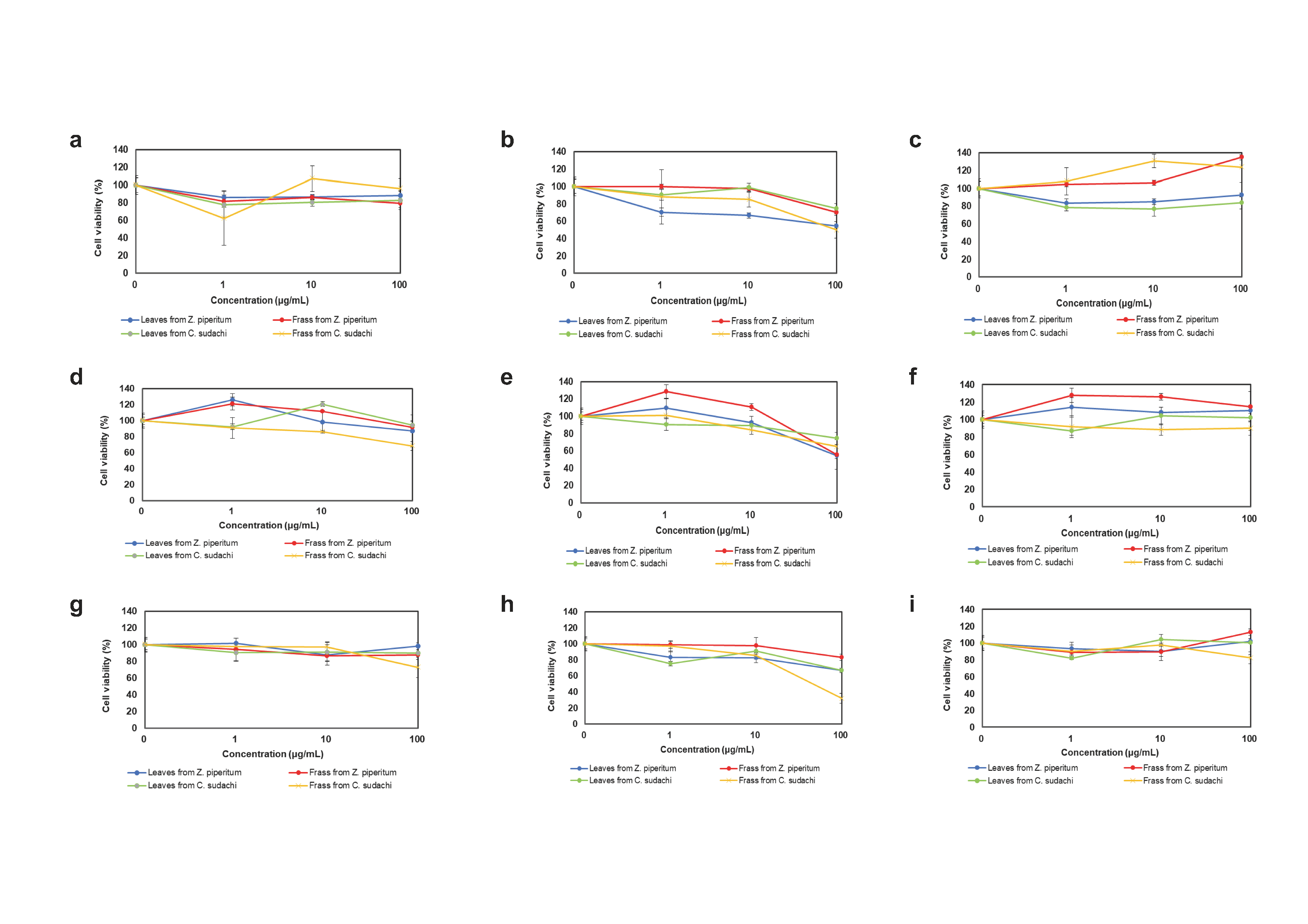

Supplement: Supplementary file 6 — Supplementary Figure 2. [file 41598_2020_74590_MOESM6_ESM.tiff]

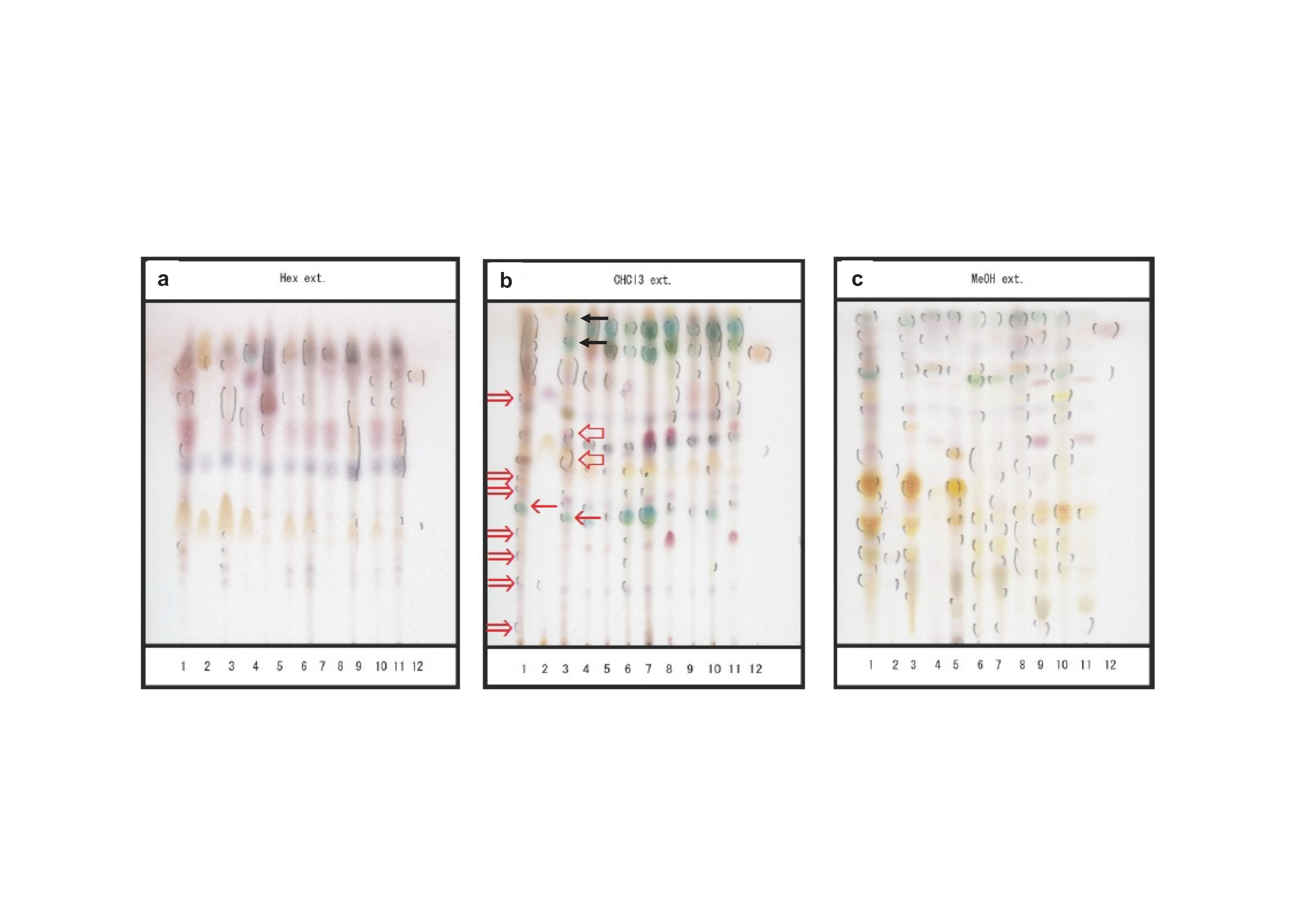

Supplement: Supplementary file 7 — Supplementary Figure 3. [file 41598_2020_74590_MOESM7_ESM.tiff]

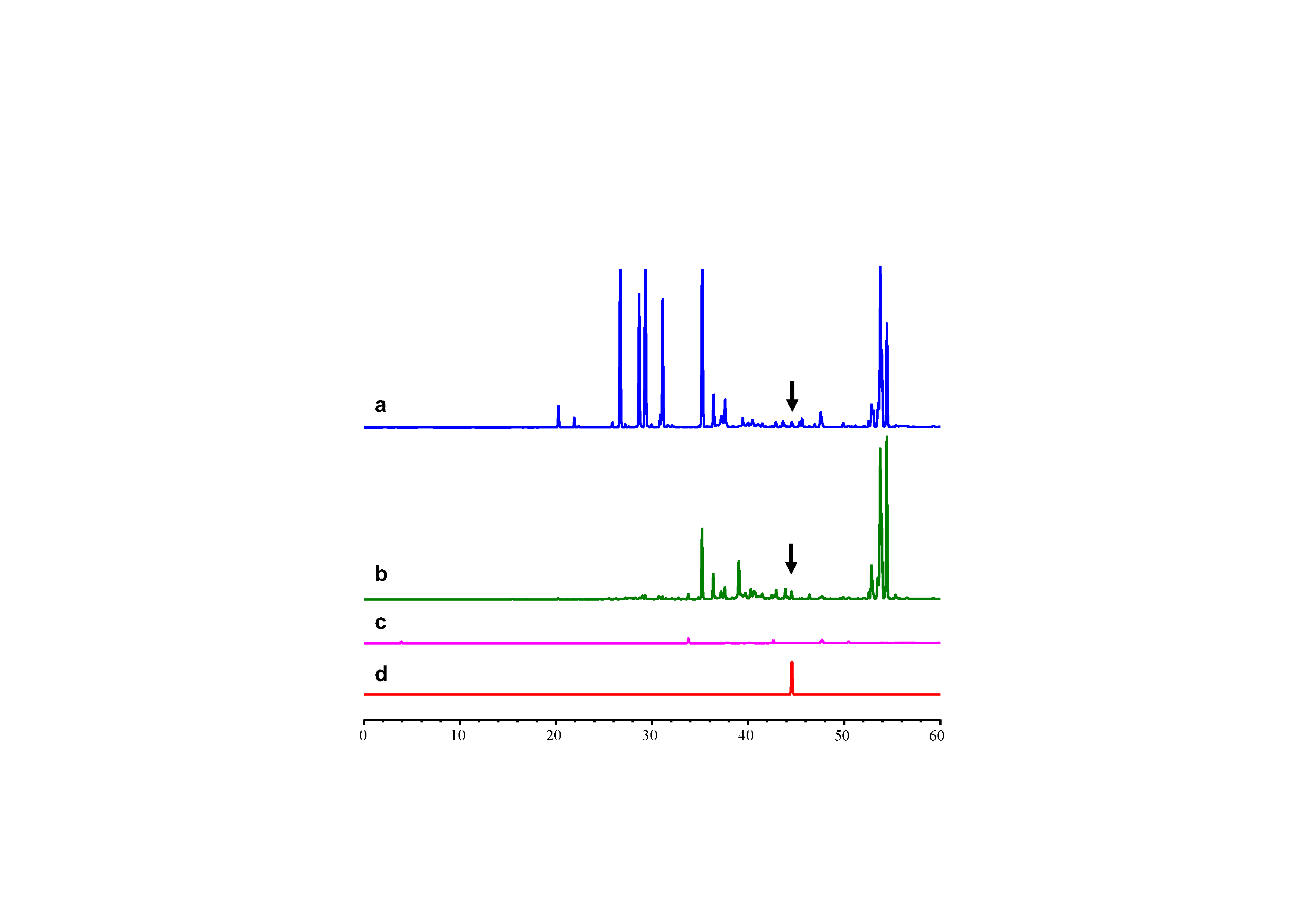

Supplement: Supplementary file 8 — Supplementary Figure 4. [file 41598_2020_74590_MOESM8_ESM.tiff]
